# Viperin, an IFN-Stimulated Protein, Delays Rotavirus Release by Inhibiting Non-Structural Protein 4 (NSP4)-Induced Intrinsic Apoptosis

**DOI:** 10.3390/v13071324

**Published:** 2021-07-08

**Authors:** Rakesh Sarkar, Satabdi Nandi, Mahadeb Lo, Animesh Gope, Mamta Chawla-Sarkar

**Affiliations:** Division of Virology, National Institute of Cholera and Enteric Diseases, P-33, C.I.T. Road Scheme-XM, Beliaghata, Kolkata 700010, India; rakeshsarkar133@gmail.com (R.S.); satabdi21@gmail.com (S.N.); mahadeblo1992@gmail.com (M.L.); gopeanimesh8reya@gmail.com (A.G.)

**Keywords:** rotavirus, interferon stimulated genes (ISGs), viperin, non-structural protein 4 (NSP4), apoptosis

## Abstract

Viral infections lead to expeditious activation of the host’s innate immune responses, most importantly the interferon (IFN) response, which manifests a network of interferon-stimulated genes (ISGs) that constrain escalating virus replication by fashioning an ill-disposed environment. Interestingly, most viruses, including rotavirus, have evolved numerous strategies to evade or subvert host immune responses to establish successful infection. Several studies have documented the induction of ISGs during rotavirus infection. In this study, we evaluated the induction and antiviral potential of viperin, an ISG, during rotavirus infection. We observed that rotavirus infection, in a stain independent manner, resulted in progressive upregulation of viperin at increasing time points post-infection. Knockdown of viperin had no significant consequence on the production of total infectious virus particles. Interestingly, substantial escalation in progeny virus release was observed upon viperin knockdown, suggesting the antagonistic role of viperin in rotavirus release. Subsequent studies unveiled that RV-NSP4 triggered relocalization of viperin from the ER, the normal residence of viperin, to mitochondria during infection. Furthermore, mitochondrial translocation of NSP4 was found to be impeded by viperin, leading to abridged cytosolic release of Cyt c and subsequent inhibition of intrinsic apoptosis. Additionally, co-immunoprecipitation studies revealed that viperin associated with NSP4 through regions including both its radical SAM domain and its C-terminal domain. Collectively, the present study demonstrated the role of viperin in restricting rotavirus egress from infected host cells by modulating NSP4 mediated apoptosis, highlighting a novel mechanism behind viperin’s antiviral action in addition to the intricacy of viperin–virus interaction.

## 1. Introduction

Rotaviruses (RVs) are the foremost etiologic agents of severe diarrhea (>200,000 deaths annually) among infants and children (<5 years) around the world [1]. It is a non-enveloped, icosahedral virus with 11 double-stranded RNA segments encoding six structural (VP1 to VP4, VP6 and VP7) and six non-structural (NSP1 to NSP6) proteins [2,3]. Structural proteins are directly involved in the formation of virion structures, whereas non-structural proteins are essential to shut off host protein synthesis [4,5,6], to evade host immune responses [7] and to form the cytosolic virus replication compartment called the viroplasm [8,9,10,11]. Non-structural proteins NSP2 and NSP5 have been reported to play a pivotal role in the formation of the viroplasm, where early stages of viral morphogenesis such as viral RNA replication and the assembly of double-layered particles (DLPs) take place [8,12]. Partially assembled DLPs, composed of 11 dsRNA segments surrounded by the inner shell of VP2 and the outer shell of VP6, are released from the viroplasm and receive their outer layer, composed of VP4 and VP7, in the rough endoplasmic reticulum (RER), forming triple-layered particles (TLPs) [13]. Finally, matured infectious TLPs are released from the infected host cells by either lytic or non-lytic mechanisms [14].

Rotaviral non-structural protein 4 (NSP4), encoded by dsRNA segment 10, is a 175 amino acid-long protein having a fundamental role in both viral morphogenesis and pathogenesis. It was initially identified as the first viral enterotoxin that could induce diarrhea in young mice in both age- and dose-dependent manners via calcium-dependent signalling pathways. NSP4 is an ER membrane glycoprotein that alters the intracellular calcium homeostasis of rotavirus-infected cells through increasing cytosolic Ca^2+^ concentration by forming aqueous pores in the ER membrane through its viroporin domain [15,16]. This NSP4-induced elevation of cytoplasmic [Ca^2+^] regulates the formation of the viroplasm and also initiates the early stages of autophagy. NSP5, a key component of the viroplasm, has two pseudo-EF-hand Ca^2+^ binding sites. Binding of Ca^2+^ to these sites triggers the aggregation of soluble NSP5 into viroplasm-like components [17]. Though autophagy maturation is inhibited, the early stages of autophagy are required for the heightened production of infectious rotavirus particles [18,19]. Being an ER membrane protein, NSP4 acts as an intracellular receptor for DLP during the entry of this subviral particle into the ER, where DLP receives the outermost layer and converts to TLP [20,21]. NSP4 has also been reported to be involved in recruiting the fission active pool of Ser161-pDrp1 to the mitochondria, leading to augmented mitochondrial fission during RV infection [22].

In the never-ending tug-of-war between viruses and their hosts, both have evolved mechanisms to counteract one another. Essential to establishing the host’s antiviral state, interferon (IFN) production by rotavirus-infected intestinal epithelial cells is primarily regulated by two RLRs (RIG-I like receptors), namely RIG-I and MDA-5 (melanoma differentiation-associated protein 5) [23]. Recognition of rotavirus PAMPs (pathogen-associated molecular patterns), most likely dsRNA, by RIG-I (retinoic acid inducible gene 1) and/or MDA-5, leads to the activation of transcription factors IRF3, IRF7 and NF-kB, which translocate into the nucleus and activate transcription of IFN. Consecutively, viruses have evolved numerous strategies to combat IFN response in order to establish successful replication. Rotavirus is no exemption, with numerous reports showing that rotaviral non-structural protein 1 (NSP1) manages to antagonize the optimized expression of IFN-α/β by adopting several countermeasures. These countermeasures include the avoidance of nuclear accumulation of activated STAT1/2 (signal transducers and activators of transcription) [24] and the proteasome-mediated degradation of β-TrCP, an important F-box substrate recognition protein required to activate NF-kB [25] and members of the IRF family including IRF3, IRF5 and IRF7 [26]. These counterstrokes taken by NSP1 to confine the IFN response emphasize the importance of that response in combating rotavirus replication and pathogenesis. Despite the cumulative antagonistic effect of NSP1 to inhibit IFN secretion, many in vitro and in vivo studies have shown upregulation of IFN-α/β during rotavirus infection [23,27]. A microarray study from our group also confirmed the induction of various interferon-stimulated genes (ISGs) during rotavirus infection in vitro [28], suggesting IFN plays an important role in limiting rotavirus replication. The protective effects of type I and III IFNs against rotavirus infection are possibly mediated through the induction of ISGs with antiviral properties. To date, the specific anti-viral mechanism of few ISGs have been addressed in the course of rotavirus infection.

Viperin (virus inhibitory protein, endoplasmic reticulum-associated, interferon inducible), also known as Cig5 (cytomegalovirus-induced gene 5 protein) or RSAD2 (radical S-adenosyl methionine domain-containing protein 2) is an ISG that is induced by both the type I and type II IFN response pathways. Viperin was initially identified in human fibroblasts infected with human cytomegalovirus (HCMV) [29,30], and later in other viruses, namely the sendai virus (SV) [31], influenza virus [32], vesicular stomatitis virus (VSV) [33], pseudorabies virus [33], japanese encephalitis virus (JEV), sindbis virus (SINV) [34], west nile virus (WNV) [35], hepatitis C virus (HCV) [36,37,38], chikungunya virus (CHIKV) [39,40], rhinovirus [41], yellow fever virus (YFV) [42], lymphocytic choriomeningitis virus (LCMV) [43], human immunodeficiency virus (HIV) [44], dengue virus (DENV) [45], measles virus (MV) [46], zika virus (ZV) [47,48], herpes simplex virus 1 (HSV-1) [49,50] and rotavirus (RV) [28] have also been reported to induce viperin.

Viperin, a 42-kDa protein of the radical SAM family of enzymes, is composed of three distinct domains: the N-terminal domain, a highly conserved radical SAM domain and a C-terminal domain [51]. The N-terminal domain contains an amphipathic helix that is known for its ability to bind viperin to the cytosolic face of the ER membrane. The radical SAM domain contains the four conserved motifs CX_3_CX_2_C, GGE, SNG and ISCDS, which are known to be common among many other members of the radical SAM family of enzymes. The CX_3_CX_2_C motif is involved in binding with the [4Fe-4S] cluster, while the GGE, SNG and ISCDS motifs all appear to function in the binding of SAM. This radical SAM domain catalyzes the conversion of SAM to methionine and a 5′ deoxyadenosyl radical, (5′-dAdo·) which subsequently perform a variety of radical mediated enzymatic reactions such as sulfur insertions, hydrogen abstractions, rearrangements, methylation reactions, DNA repair and cofactor biosynthesis [52,53,54,55,56]. The C-terminal domain displays conspicuous resemblances across species and has been described as having antiviral function against numerous viruses [51]. Though the antiviral effect of viperin has been well established in different viruses (HCMV, influenza, JEV, DENV-2, HCV, HIV, MV and ZV), the specific underlying mechanisms remain largely unknown [29,30,31,32,33,34,35,36,37,38,44,45,46,47,48]. However, a recent study demonstrated the anti-viral action of viperin’s SAM domain, which catalyzed the conversion of cytidine triphosphate (CTP) to 3′-deoxy-3′,4′-didehydro-CTP (ddhCTP), which acted as a chain terminator for the RNA-dependent RNA polymerases from multiple members of the Flavivirus genus such as zika virus [57].

In a microarray study by our group to assess the induction of genes following rotavirus infection, 4.14-fold induction of viperin was observed in RV-SA11-infected HT-29 cells [28]. Thus, the present study was undertaken to delineate the role of viperin during the rotavirus replication cycle. Our data showed that rotavirus infection resulted in the increased expression of the viperin protein in a strain-independent manner. Viperin was found to restrict rotavirus release from the infected host cells, but it had no significant effect on total infectious virus production. Mechanistic studies unveiled that RV-NSP4 relocalized viperin from the ER to mitochondria where viperin, in turn, inhibited mitochondrial translocation of NSP4, leading to the reduced release of Cyt c into the cytosol and restricted activation of the intrinsic apoptosis pathway.

## 2. Materials and Methods

### 2.1. Cell Culture and Virus Infection

Cell culture-adapted rotavirus strains such as simian strain SA11 (G3P [2]), human strains Wa (G1P [8]) and KU (G1P [8]) and bovine strain A5-13 (G8P [1]) were used for this study. Human embryonic kidney cell line HEK293 (ATCC number: CRL-1573™) was cultured in minimal essential medium (MEM) and human intestinal epithelial (HT29) cells and Vero cells were cultured in Dulbecco’s modified Eagle’s medium (DMEM) supplemented with US-certified 10% fetal bovine serum and 1% antibiotic–antimycotic solution (Invitrogen, Carlsbad, CA, USA). Cells were maintained in a 5% CO_2_ at 37 °C humidified incubator. For infection, viruses were activated with acetylated trypsin (10 μg/mL) at 37 °C for 30 min, diluted as required by the multiplicity of infection (MOI) and added to the cells for adsorption (45 min) at 37 °C, followed by three washes with media to remove the unbound virus. Infection was continued in fresh medium. All the studies were performed at a MOI of 3, and the time of virus addition was considered 0 h post infection (hpi).

### 2.2. Reagents and Antibodies

A protease inhibitor cocktail (P2741), a phosphatase inhibitor cocktail (P5726), pan caspase inhibitor Z-VAD-FMK (V116), Ca^2+^ chelator BAPTA-AM (A1076) and monoclonal antibody against FLAG epitope (SAB420007) were procured from Merck (Sigma-Aldrich, St. Louis, Missouri, USA). Both mouse monoclonal antibodies (ab107359) and rabbit polyclonal antibodies (ab73864) against viperin were purchased from Abcam, USA. Mouse monoclonal antibodies against COX-4 (sc-376731) and GAPDH (sc-47724) and rabbit polyclonal antibodies against calnexin (sc-11397) were purchased from Santa Cruz Biotechnology, USA. Rabbit polyclonal antibodies against cleaved caspase-3 (9661s) and cleaved caspase-9 (9501) were procured from Cell Signaling Technology, USA. Monoclonal antibodies against rotaviral structural protein VP6 (3R103C10) were obtained from HyTest, Finland. Monoclonal antibodies against cytochrome c (CH2.B4) were acquired from BD Biosciences. Antisera against rotaviral non-structural protein NSP4 and VP7 were raised in rabbits, according to standard protocols at the Department of Virology and Parasitology, Fujita Health University School of Medicine, Aichi, Japan.

### 2.3. Western Blotting

Cells were harvested, washed once in phosphate-buffered saline, and lysed in Totex buffer (20 mM HEPES at pH 7.9, 0.35 M NaCl, 20% glycerol, 1% NP-40, 1 mM MgCl_2_, 0.5 mM EDTA, 0.1 mM EGTA, 50 mM NaF and 0.3 mM Na_3_VO_4_) containing a mixture of protease and phosphatase inhibitors (Sigma-Aldrich, St. Louis, Missouri, USA). The protein concentration of the whole cell lysates was measured by the Bradford method, and an equal amount of protein from each sample was boiled in sample buffer (final concentration: 50 mM Tris, pH 6.8, 1% SDS, 10% glycerol, 1% β-mercaptoethanol and 0.01% bromphenol blue) for 5 min. Boiled cell lysates were separated by SDS-PAGE and transferred to polyvinylidene difluoride (PVDF) membranes (Millipore) and then probed with the indicated primary antibodies. Primary antibodies were detected using horseradish peroxidase-conjugated secondary antibodies (Pierce, Rockford, IL, USA) and chemiluminescent substrate (Millipore, Billerica, MA, USA). Blots were re-probed with anti-GAPDH antibodies to confirm equal protein loading. All experiments were done three times, from which, one representative blot is given in the figure.

### 2.4. Real-Time PCR

Total RNA was extracted from HT-29 cells, transfected with either cont-shRNA or viperin-shRNA, using TRIzol (Invitrogen, Grand Island, NY, USA), according to the manufacturer’s instructions. Next, 500 ng of RNA from each sample were used to prepare cDNA, using the Superscript II reverse transcriptase (Invitrogen) with random hexamer primers by incubating at 42 °C for 1 h. Real-time PCR reactions (50 °C, 2 min; 95 °C, 10 min; 40 cycles of 95 °C, 15 s and 60 °C, 30 s; and 72 °C, 10 min) were performed in triplicate using SYBR Green intercalating dye (Applied Biosystems, Foster City, CA, USA) with primers specific for *vp6* (FP:5′-CAGTGATTCTCAGGCCGAATA-3′; RP: 5′-GGCGAGTACAGACTCACAAA-3′) and *gapdh* (FP: 5′-GTCAACGGATTTGGTCGTATTG-3′; RP: 5′-TGGAAGATGGTGATGGGATTT-3′) in Step One Plus (Applied Biosystems). The viral gene expressions were normalized to the *gapdh* transcript, using the formula 2^−ΔCT^ (ΔC_T_ = C_T vip-shRNA_-C_T cont-shRNA_), where C_T_ was the threshold cycle and data was represented as “relative fold change of viral transcript compared to GAPDH transcript”. Each bar denoted the mean fold chance ± SD of three independent experiments. The *p* values were calculated using an unpaired Student’s t test.

### 2.5. Cloning of Viperin and NSP4

Full-length human viperin (Accession No. NM_080657) and region-specific mutants of viperin were cloned in pFLAG-CMV6b expression vector (Sigma). Full-length NSP4 of RV-SA11 (Accession No. DQ838625) was cloned in pcDNA^TM^6/V5-His B expression vector (Invitrogen). Specific primers used for cloning are given in Table 1. To prepare the vector expressing the full-length viperin and region-specific mutants, HT29 cells were stimulated with interferon β, followed by RNA extraction using TRIzol LS reagent (Invitrogen). To prepare vectors expressing the full-length NSP4 of RV strain SA11 H[96], viral RNA was extracted from RV-SA11-infected HT-29 cells. Subsequently, cDNA was prepared from RNA by reverse transcription (RT)-PCR, followed by PCR with the respective primer sets and cloning into specific vectors.

### 2.6. Knockdown of Viperin

The DNA sequence encoding short hairpin RNA (shRNA) specific for viperin was generated by annealing the forward and reverse oligos and cloned into pLKO.1-TCR cloning vector as per the instructions mentioned at the Addgene site (https://www.addgene.org/protocols/plko/; accessed on 20 December 2018). Forward and reverse oligos (forward oligo: 5′-CCGGAAGTGTTCCAGTGCCTCTTAACTCGAGTTAAGAGGCACTGGAACACTTTTTTTG-3′; reverse oligo: 5′-AATTCAAAAAAAGTGTTCCAGTGCCTCTTAACTCGAGTTAAGAGGCACTGGAACACTT-3′) were designed using the siRNA selection program (http://sirna.wi.mit.edu/; accessed on 10 November 2018) hosted by the Whitehead Institute for Biomedical Research. pLKO.1-TRC vector-encoding shRNA against luciferase (cont-shRNA) was used as the negative control. pLKO.1-TRC cloning vector [58] was a gift from David Root (Addgene plasmid# 10878). Transfection of viperin-shRNA and control-shRNA were carried out using Lipofectamine 2000 (Invitrogen) reagent as per the manufacturer’s instructions. The knockdown efficiency of viperin-shRNA was evaluated by western blot using viperin-specific antibodies.

### 2.7. Confocal Microscopy

HT-29 cells grown on a coverslip (30–50% confluency) were infected with RV-SA11, followed by fixation with 4% paraformaldehyde (in PBS) at 4 °C for 20 min. Next, cells were washed with PBS 4–5 times and permeabilized with PBS supplemented with 0.1% Triton X-100 (*v*/*v*) for 30 min. Samples were then incubated in blocking solution (PBS supplemented with 2% BSA) for 1 h at room temperature. Next, cells were washed with cold PBS and incubated overnight with primary antibodies, specific for viperin (raised in mice) and NSP4 (raised in rabbits), at 4 °C, followed by rhodamine-conjugated anti-mouse and Dylight488-conjugated anti-rabbit secondary antibodies (Jackson Laboratories, Inc., West Grove, PA, USA) for 1 h at room temperature. Next, the coverslips were washed with PBS and mounted with 4′, 6′-diamidino-2-phenylindole (DAPI) (Vector Laboratories, Burlingame, CA, USA). Imaging was done using a Zeiss Axioplan microscope (63X oil immersion). Excitation and emission detection for each fluorophore was performed sequentially to avoid cross-talk. Acquired images were analysed using Zen Blue software. Experiments were done in triplicate, and one selected image is presented in the figure.

### 2.8. Estimation of Infectious Virus Particle by Plaque Assay

Estimation of infectious virus particles was performed by plaque assay. Briefly, monolayers of MA104 cells grown in six-well plates were infected with serial dilutions (10^2^ to 10^8^) of viral supernatants. After 45 min of adsorption, the inoculum was removed, and the cells were overlaid with 0.7% agar in 1X MEM with 1 μg of trypsin/mL. At 36–48 h post-infection, a second agar overlay (0.7% agar in 1X MEM with 0.1% neutral red) was added, and the plates were incubated at 37 °C until the plaques were visualized. Viral plaque forming unit (PFU) was calculated as PFU/mL (Original stock) = ((1/dilution factor) × (number of plaques) × (1/[ml of inoculum/plate])) [59].

### 2.9. Isolation of the ER Fraction

The ER fraction of infected or transfected HT-29 cells was isolated by the Endoplasmic Reticulum Isolation Kit (ER0100, Sigma-Aldrich), using the manufacturer’s instructions.

### 2.10. Isolation of Mitochondrial and Cytosolic Fractions

Mitochondria were isolated from infected or transfected HT-29 cells by the differential centrifugation method. Cells were trypsinized and washed with cold PBS. Cells were re-suspended in freshly prepared cold isolation buffer (0.3 M mannitol, 0.1% BSA, 0.2 mM EDTA, 10 mM HEPES, pH 7.4 with KOH, supplemented with 1X protease inhibitor cocktail) and homogenized on ice followed by centrifugation (1000× *g* at 4 °C) for 10 min. Supernatants were carefully collected in a separate tube and further centrifuged at 7000× *g* for 15 min at 4 °C. The final supernatant was saved as the cytosolic fraction. The pellets representing the mitochondrial fraction were washed with cold wash buffer (0.25 M sucrose and 10 mM HEPES, pH 7.5), followed by centrifugation at 7000× *g* for 10 min. The pellets were stored at −80 °C in a freezer unless used immediately. Mitochondrial proteins were extracted by re-suspending the mitochondrial pellets in a buffer containing 7 M urea, 2 M thiourea, 4% CHAPS, 120 mM dithiothreitol (DTT), 2% ampholytes (pH 3–10) and 40 mM tris HCl (pH 5). Pure mitochondrial fractions were isolated by ultracentrifugation using iodixanol as described previously [60].

### 2.11. Co-Immunoprecipitation

Infected or transfected cells were lysed (lysis buffer: 0.025 M Tris, 0.15 M NaCl, 0.001 M EDTA, 1% NP-40, 5% glycerol; pH 7.4) and pre-cleared by incubating with protein A-Sepharose at 4 °C for 2 h. Next, cell lysates were incubated with specific antibodies overnight at 4 °C, followed by incubation with protein A-Sepharose beads (GE Healthcare, Uppsala, Sweden) for 4 h. The beads were washed five times with 1 X lysis buffer, and bound proteins were separated by SDS-PAGE (10%) and transferred to a PVDF membrane. Subsequently, western blot analysis was performed to detect the presence of specific proteins in the immunoprecipitate.

### 2.12. MTT Assay

After specific treatment (mentioned in the results section), HT-29/HEK-293 cells were incubated for 72 h and subsequently, cell viability was measured by MTT assay. Briefly, MTT solution was added to the cells at a final concentration of 0.5 mg/mL and incubated at 37 °C for 4 h. The purple formazan crystals formed after incubation were completely dissolved in 200 μL MTT solvent (4 mM HCl, 0.1% Nonidet P-40 in isopropanol). Subsequently, the optical density (OD) of the solution was measured at 570 nm, and cell viability was calculated using the formula (OD_Sample_-OD_Blank_) × 100/(OD_Control_-OD_Blank_). Data was represented as “cell viability (%)”, considering the cellular viability of untreated control cells as 100%.

### 2.13. Statistical Analysis

Data were expressed as mean ± the standard deviation (SD) of at least three independent experiments (*n* ≥ 3). Statistical significance was analyzed by the unpaired Student’s t test. In all experiments, *p* ≤ 0.05 was considered statistically significant.

## 3. Results

### 3.1. Rotavirus Infection Induced the Expression of Viperin

**Figure 1:**
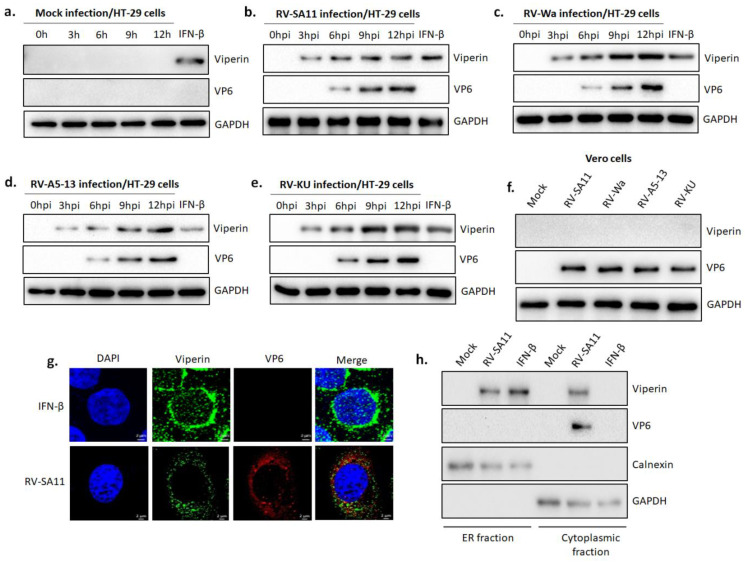
Viperin expression was triggered during RV infection. HT-29 cells were either (**a**) mock infected or infected with (**b**) RV-SA11, (**c**) RV-Wa, (**d**) RV-A5-13 or (**e**) RV-KU at an MOI of 3 and incubated until the indicated time points post-infection. Next, cell lysates were prepared and subjected to SDS-PAGE/western blot analysis using antibodies specific for viperin, VP6 and GAPDH. VP6 and GAPDH served the purpose of the infection marker and the internal loading control, respectively. (**f**) Vero cells, attenuated for type-I IFN production, were either mock infected or infected with RV-SA11, RV-Wa, RV-A5-13 or RV-KU at an MOI of 3 and incubated for 12 hpi. Subsequently, viperin expression was assessed in whole cell lysates by western blot using anti-viperin, anti-VP6 and anti-GAPDH antibodies. (**g**) HT-29 cells were either infected with RV-SA11 for 9 h or treated with IFN-β for 6 h. Next, cells were fixed, permeabilized and stained with primary antibodies specific for viperin and VP6. Secondary staining was performed with Rhodamin-labeled anti-mouse (for VP6) and Dylight488-labeled anti-rabbit (for viperin) antibodies. DAPI was used for mounting. Imaging was performed with a confocal microscope (63X oil immersion). Images were processed with Zen Blue software. Scale bar, 2 μm. (**h**) ER and cytosolic fractions of HT-29 cells, either mock infected or infected with RV-SA11 for 9 h, were subjected to SDS-PAGE/western blot analysis to evaluate the expression of viperin, VP6, calnexin and GAPDH. Calnexin and GAPDH served the purpose of the ER marker and the cytoplasmic marker, respectively.

Viperin, a well-documented antiviral protein, has previously been reported to be induced in the course of infection of numerous viruses. To investigate whether viperin was induced during rotavirus infection, we used western blot analysis to evaluate viperin expression in the lysates of HT-29 cells, either mock-infected or infected with RV-SA11 (MOI 3) at different hours post-infection (0 hpi, 3 hpi, 6 hpi, 9 hpi and 12 hpi). Parallelly, the lysate of HT-29 cells treated with IFN-β for 6 h was used as a positive control for viperin expression. Results showed null expression of viperin in mock-infected HT-29 cells (Figure 1a), whereas viperin expression was detected in RV-SA11-infected HT-29 cells as early as 3 hpi and progressively intensified with increasing time points (Figure 1b), suggesting that rotavirus infection induced heightened expression of viperin. To this end, viperin expression was also evaluated in HT-29 cells infected with different rotavirus strains of both human (Wa and KU) and bovine (A5-13) origin. Consistently, viperin induction was observed post-RV-Wa (human strain), -RV-A5-13 (bovine strain) or -RV-KU (human strain) infection (Figure 1c–e), suggesting that rotavirus-induced viperin expression was a strain-independent phenomenon. Although viperin is a well-known interferon-stimulated protein, some viruses have been shown to directly induce viperin by the IFN-independent pathway. To check whether the regulation of viperin expression during RV infection was umpired by the IFN-dependent pathway, Vero cells, attenuated for type-I IFN production, were infected with different RV strains (SA11, Wa, A5-13, KU) at an MOI of 3, and viperin expression was assessed at 12 hpi. Viperin expression was not witnessed in Vero cells infected with any of the four RV strains (Figure 1f), demonstrating the probable association of the type-I IFN-dependent pathway in inducing viperin during RV infection. Viperin is an ER membrane-resident protein. We used confocal microscopy to investigate the intracellular localization of RV-induced viperin. HT-29 cells treated with IFN-β were used as a positive control. Viperin was found to disperse throughout the cytoplasm of RV-SA11-infected HT-29 cells, whereas it was only restricted to the perinuclear regions in IFN-β treated cells, suggesting the altered localization of viperin in RV-infected cells (Figure 1g). Furthermore, we performed western blot analysis of the ER and cytoplasmic fractions of RV-SA11-infected or IFN-β-treated HT-29 cells to confirm subcellular localization of viperin. Viperin was only found in the ER fraction of IFN-β treated cells, while in RV-SA11 infected cells, it was observed in both the ER and cytoplasmic fractions, suggesting RV infection might redirect viperin localization from the ER to the cytosol (Figure 1h). Conjointly, these data suggested that rotavirus infection induced viperin expression by the type-I IFN-dependent pathway and redirected viperin localization from the ER.

### 3.2. Viperin Delayed the Release of Rotavirus from the Infected Host Cell

**Figure 2:**
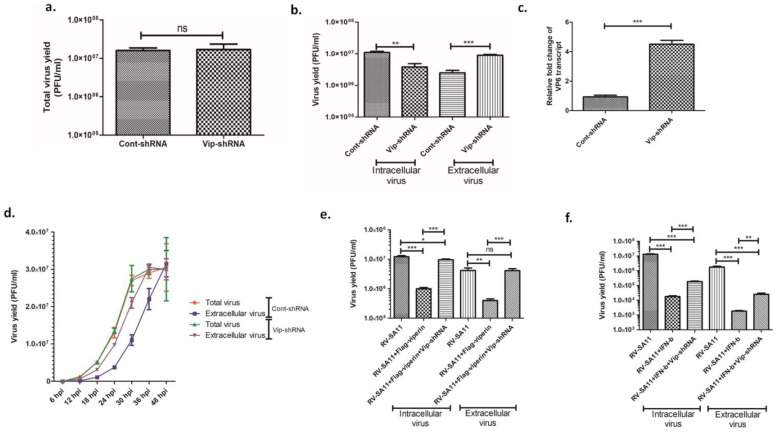
RV release was facilitated in viperin-knocked-down cells (**a**) HT-29 cells, pre-transfected with either cont-shRNA or vip-shRNA, were infected with RV-SA11 (MOI 3) and incubated for 24 hpi. Next, cells were collected along with extracellular medium, freeze–thawed three times, centrifuged and subjected to plaque assay to measure viral titer. Data represent mean virus particles ± SD of three independent experiments (ns: not significant, unpaired Student’s *t*-test). (**b**) HT-29 cells, pre-transfected with either cont-shRNA or vip-shRNA, were infected with RV-SA11 (MOI 3) for 24 hpi. After incubation, extracellular medium was collected and used to quantify released viral particles by plaque assay whereas infected cells were collected in fresh medium, freeze–thawed three times, centrifuged and subjected to plaque assay to measure intracellular viral titer. Data represent mean virus particles ± SD of three independent experiments; *** represents *p* ≤ 0.001, ** represents *p* ≤ 0.01, unpaired Student’s *t*-test. (**c**) HT-29 cells transfected with either cont-shRNA or vip-shRNA were infected with RV-SA11 at an MOI of 3 and incubated for 24 h. After incubation, extracellular medium was collected, clarified and treated with RNAse. Next, RNA was isolated from the medium and used to quantify the level of VP6 RNA by real-time PCR. Data represent mean fold change ± SD of three independent experiments; *** represents *p* ≤ 0.001, unpaired Student’s *t*-test. (**d**) HT-29 cells, pre-transfected with either cont-shRNA or vip-shRNA, were infected with RV-SA11 (MOI 3) and incubated until the indicated time points. After incubation, both the total infectious viral titer and released viral titer were measured by plaque assay. Data represent mean viral titer ± SD of three independent experiments. (**e**) RV-SA11-infected Vero cells, transfected with cont-vector and cont-shRNA, FLAG-viperin and cont-shRNA or FLAG-viperin and vip-shRNA, were used to measure both the intracellular and extracellular viral particles by plaque assay. Data represent mean viral particles ± SD of three independent experiments; *** represents *p* ≤ 0.001, ** represents *p* ≤ 0.01, * represents *p* ≤ 0.05, ns represents *p* value non-significant, unpaired Student’s *t*-test. (**f**) HT-29 cells, transfected with either cont-shRNA or vip-shRNA, were treated with IFN-β for 2 h followed by infection with RV-SA11 at an MOI of 3 for 24 hpi. Next, intracellular and extracellular viruses were collected and subjected to plaque assay. Data represent mean virus particles ± SD of three independent experiments; *** represents *p* ≤ 0.001, ** represents *p* ≤ 0.01, unpaired Student’s *t*-test.

The antiviral activity of viperin against diverse groups of viral infections has been well-established. To assess the relevance of viperin induction in RV replication, HT-29 cells, transfected with either cont-shRNA or vip-shRNA, were infected with RV-SA11 at a MOI of 3, and total infectious virus (both extracellular and intracellular) production was measured at 24 hpi by plaque assay. Estimation of virus yield by plaque assay revealed viperin knockdown had no significant impact on the production of total infectious virus particles at 24 hpi (Figure 2a). Interestingly, a significant escalation (3.68 ± 0.63-fold) in extracellular infectious virus and a comparable reduction (2.97 ± 0.64 fold) in intracellular infectious virus were observed in viperin-knocked-down HT-29 cells compared to viperin-expressing HT-29 cells at 24 hpi (Figure 2b). Nevertheless, vip-shRNA had no significant effect on cell viability (Figure S2). These data suggested viperin had no effect on viral morphogenesis and possibly inhibited rotavirus release from the infected host cells. We also quantified the level of viral RNA in the clarified and RNAse-treated supernatant of RV-SA11-infected HT-29 cells, transfected with either cont-shRNA or vip-shRNA, at 24 hpi. Results showed a 4.5-fold increase of VP6 RNA in the extracellular medium of viperin-silencing cells compared to viperin-expressing cells at 24 hpi (Figure 2c), which was consistent with increased viral titer in the extracellular medium of viperin-silencing cells. We further performed a kinetic study of total virus formation and extracellular viral release in RV-SA11-infected HT-29 cells transfected with either cont-shRNA or vip-shRNA to ensure whether viperin restricted or delayed rotavirus release (Figure 2d). The kinetic study showed viperin silencing had no effect on total virus production, which reached a plateau at 36 hpi. This result was consistent with previous observations. However, viperin knockdown resulted in delayed kinetics of rotavirus release compared to viperin-expressing cells. Notably, the extracellular viral titer attained the level of total viral titer at 36 hpi in cells transfected with vip-shRNA, whereas it took 48 h for viperin-expressing cells (Figure 2d). To further substantiate the results, the intracellular and extracellular viral titer of RV-SA11-infected Vero cells (deficient in IFN production and, thus, viperin expression) overexpressing either viperin or both viperin and vip-shRNA were assessed by plaque assay at 24 hpi. Results showed 10.59-fold reduction of viral titer in the extracellular medium of viperin-overexpressing Vero cells compared to the viperin non-overexpressing cells. As expected, silencing of viperin by shRNA inhibited the viperin-restricted release of rotavirus in the extracellular medium (Figure 2e). The protective role of IFN-β in the release of rotavirus was also found to be moderately compromised in the viperin-knocked-down HT-29 cells (Figure 2f). Collectively, these results confirmed the role of viperin in modulating rotavirus egress from the infected host cells.

### 3.3. Full-Length Viperin Was Essential for Anti-Rotaviral Function

**Figure 3:**
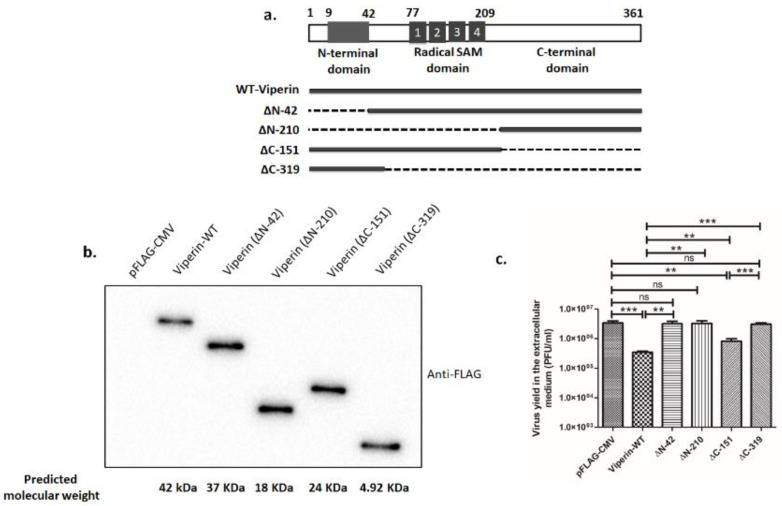
Full-length viperin was indispensable to prevent rotaviral egress from the infected cells. (**a**) Schematic representation of the structures of the viperin and its mutants used in the study. Residues 9–42 constituted the N-terminal amphipathic α-helix (depicted in gray), which was required for its localization to the cytosolic face of the ER and lipid droplets. The radical SAM domain (residues 77-209) containing four conserved motifs (marked in gray) was essential for its functional activities. The conserved C-terminus was involved in dimerization, but no other functions have yet been ascribed to it. (**b**) Expressions of the FLAG-tagged wild-type viperin and its four mutants in HEK-293 cells. (**c**) HEK-293 cells overexpressing either wild-type viperin or viperin mutants were infected with RV-SA11 at an MOI of 3 and incubated for 24 h. After incubation, extracellular mediums were collected and used to measure viral yield by plaque assay. Data represent mean virus particles ± SD of three independent experiments; *** represents *p* ≤ 0.001, ** represents *p* ≤ 0.01, ns represents *p* value non-significant, unpaired Student’s *t*-test.

In order to identify the antiviral domain of viperin, FLAG-tagged, wild-type viperin and four deletion mutants, namely ∆N-42 (lacking an N-terminal domain), ∆N-210 (lacking both an N-terminal domain and a radical SAM domain), ∆C-151 (lacking a C-terminal domain) and ∆C-319 (lacking both a radical SAM domain and a C-terminal domain) were constructed (Figure 3a). Expression of these constructs was confirmed in HEK-293 cells by immunoblotting with anti-FLAG antibody, following overexpression (Figure 3b). We also found overexpression of these constructs had no significant effects on cell viability (Figure S3). To analyze the antiviral effect of these deletion mutants, HEK-293 cells overexpressing either viperin-WT or mutant viperin were infected with RV-SA11 (MOI 3). Quantification of the released rotavirus particles at 24 hpi showed that deletion of either the N-terminal domain (∆N-42) or both the N-terminal domain and the radical SAM domain (∆N-210) resulted in complete abrogation of the antiviral effects of viperin, suggesting that the N-terminal domain was essential for imparting the antiviral function of viperin (Figure 3c). Furthermore, deletion of the C-terminal domain (∆C-151) showed a partial increase in rotavirus release compared to the wild-type viperin (viperin-WT). However, deletion of both the C-terminal domain and the radical SAM domain (∆C-319) resulted in complete abrogation of the antiviral function of viperin and showed comparable release of rotavirus particles as control vector-transfected cells (Figure 3c). Collectively, these data suggest that deletion of either the N-terminal domain or both the SAM domain and the C-terminal domain could completely diminish the antiviral action of viperin, suggesting that full-length viperin is essential to perform antiviral actions.

### 3.4. NSP4 Triggered Viperin Relocalization from the ER to the Mitochondria during Rotavirus Infection

**Figure 4:**
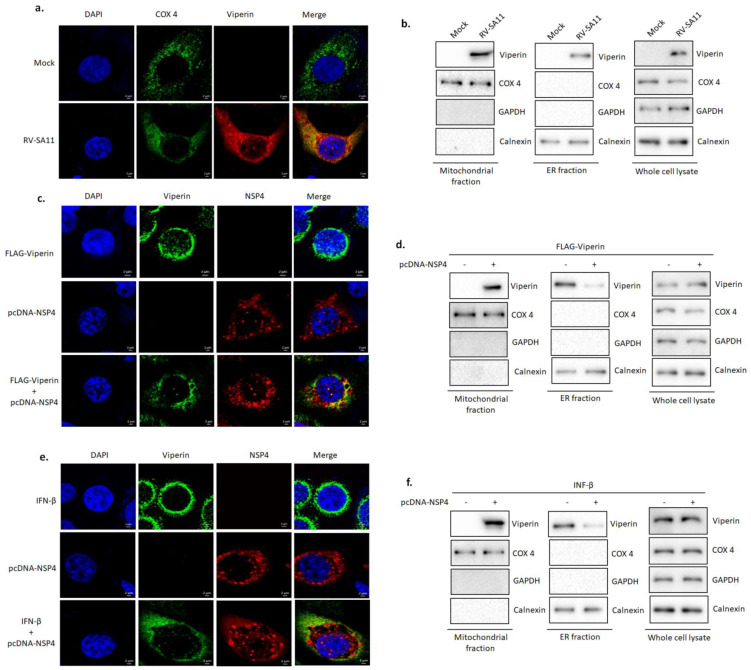
Viperin was relocalized from the ER to the mitochondria, triggered by RV-NSP4. (**a**) HT-29 cells were either mock infected or infected with RV-SA11 and incubated for 9 h. Next, cells were fixed, permeabilized and stained with anti-COX 4 and anti-viperin primary antibodies. Secondary staining was performed with Dylight488-labeled anti-mouse (for COX 4) and rhodamine-labeled anti-rabbit (for viperin) antibodies. DAPI was used for mounting. Imaging was done with a confocal microscope (63X oil immersion). Scale bar, 2 μm. (**b**) HT-29 cells were either mock infected or infected with RV-SA11 at an MOI of 3 for 9 h. Cells were harvested and used to prepare the mitochondrial fraction, the ER fraction and the whole-cell lysate. Next, the mitochondrial fraction, the ER fraction and the whole cell lysate were subjected to SDS-PAGE/western blot analysis to check the expressions of viperin, COX 4, calnexin and GAPDH. COX 4 and calnexin were used as the mitochondrial marker and the ER marker, respectively. GAPDH was used as the cytoplasmic marker to rule out cross contamination of the mitochondrial fraction and the ER fraction with cytosol. (**c**) HT-29 cells were transfected with FLAG-viperin, pcDNA-NSP4 or both FLAG-viperin and pcDNA-NSP4 and incubated for 36 h. Next, cells were fixed, permeabilized and stained with anti-viperin and anti-NSP4 antibodies. Secondary staining was done with Dylight488-labeled anti-mouse (for viperin) and rhodamine-labeled anti-rabbit (for NSP4) antibodies. DAPI was used for mounting. Imaging was performed with a confocal microscope (63X oil immersion). Scale bar, 2 μm. (**d**) HT-29 cells were transfected with either pcDNA-NSP4 or both pcDNA-NSP4 and FLAG-viperin and incubated for 36 h. After incubation, the mitochondrial fraction, the ER fraction and the whole cell lysate were prepared and subjected to western blot to check expressions of viperin, COX 4, calnexin and GAPDH. (**e**) HT-29 cells, either treated with IFN-β (6 h) or transfected with pcDNA-NSP4 or both transfected with pcDNA-NSP4 and treated with IFN-β were subjected to confocal microscopy as described in section c. Scale bar, 2 μm. (**f**) HT-29 cells, transfected with either pcDNA-6B or pcDNA-NSP4, were treated with IFN-β for 6 h. After incubation, the mitochondrial fraction, the ER fraction and the whole-cell lysate were prepared and subjected to western blot to check expressions of viperin, COX 4, calnexin and GAPDH.

As an initial approach to identify the potential antiviral mechanism of viperin, we questioned the specific intra-cytosolic location of viperin during rotavirus infection. Viperin has previously been reported to relocalize from the ER to the mitochondria during HCMV infection [61]. With this in mind, we investigated the subcellular localization of viperin in RV-SA11 or mock-infected HT-29 cells at 9 hpi by confocal microscopy. COX-4 was labeled as a mitochondrial marker protein. Expectedly, a considerable colocalization (Pearson’s correlation coefficient 0.92) was observed between viperin and COX-4 (Figure 4a), indicating the relocalization of viperin to the mitochondria in rotavirus-infected cells. Western blot analysis also confirmed the presence of viperin in the mitochondrial fraction of RV-SA11-infected HT-29 cells at 9 hpi (Figure 4b). Next, we sought to ascertain the viral trigger that redistributed viperin from the ER to the mitochondria. Rotaviral non-structural protein 4 (NSP4) has previously been reported to traffic in both the ER and the mitochondria, and thus the role of NSP4 in viperin relocalization was assessed. HT-29 cells overexpressing either FLAG-viperin, pcDNA-NSP4 or both FLAG-viperin and pcDNA-NSP4 were subjected to confocal microscopy to visualize the cellular localization of viperin in the presence or absence of NSP4. Confocal imaging showed that viperin was restricted to the ER in FLAG-viperin overexpressing HT-29 cells, whereas in contrast, viperin was found to relocate from the ER in FLAG-viperin and pcDNA-NSP4-co-overexpressing HT-29 cells, demonstrating the role of NSP4 in the expulsion of viperin from the ER during rotavirus infection (Figure 4c). Notably, a significant colocalization (Pearson’s correlation coefficient 0.72) was observed between NSP4 and viperin, indicating a possible association between them. We further extended our observation by investigating the presence of viperin in the mitochondrial fraction of HT-29 cells, transfected with either FLAG-viperin or both FLAG-viperin and pcDNA-NSP4, by western blot. Results of western blot analysis showed the presence of viperin in the mitochondrial fraction of HT-29 cells co-overexpressing FLAG-viperin and pcDNA-NSP4. However, despite its presence in the whole cell lysate and the ER fraction, viperin was absent in the mitochondrial fraction of HT-29 cells overexpressing only FLAG-viperin (Figure 4d). Localization of viperin from the ER to the mitochondria was also triggered in NSP4-overexpressing cells treated with IFNβ (Figure 4e,f). Collectively, these results demonstrated that NSP4 triggered the relocalization of viperin from the ER to the mitochondria in the course of rotavirus infection.

### 3.5. Viperin Associated with NSP4

**Figure 5:**
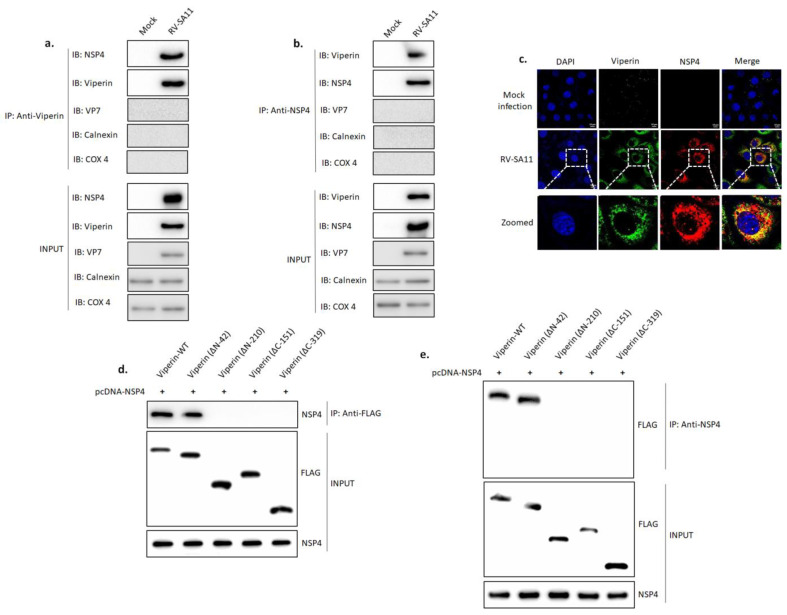
NSP4 interacted with viperin. (**a**) HT-29 cells were mock infected or infected with RV-SA11 at an MOI of 3. Cell lysates were prepared at 9 hpi and immunoprecipitated with anti-viperin antibodies, followed by western blot analysis with anti-NSP4, anti-viperin, anti-VP7, anti-calnexin and anti-COX 4 antibodies. Inputs were probed with these antibodies to confirm protein expression. (**b**) HT-29 cells were mock infected or infected with RV-SA11 at an MOI of 3. Cell lysates were prepared at 9 hpi and immunoprecipitated with anti-NSP4 antibodies, followed by western blot analysis with anti-viperin, anti-NSP4, anti-VP7, anti-calnexin and anti-COX 4 antibodies. (**c**) HT-29 cells were either mock infected or infected with RV-SA11 at an MOI of 3. After 9 h of incubation, cells were fixed, permeabilized and stained with anti-viperin and anti-NSP4 antibodies. Secondary staining was done with Dylight488-labeled anti-mouse (for viperin) and rhodamine-labeled anti-rabbit (for NSP4) antibodies. Imaging was performed with a confocal microscope (63X oil immersion). Scale bar, 10 µm. (**d**) HEK-293 cells were transfected with pcDNA-NSP4 along with FLAG-tagged wild-type viperin, ΔN42, ΔN210, ΔC151 or ΔC319 and incubated for 36 h. Next, cell lysates were prepared and immunoprecipitated with anti-FLAG antibodies. Immunoprecipitates were then subjected to western blot analysis to check for the presence of NSP4. Inputs were probed with anti-FLAG and anti-NSP4 antibodies to confirm protein expression. (**e**) HEK-293 cells were transfected with pcDNA-NSP4 along with FLAG-tagged wild type viperin, ΔN42, ΔN210, ΔC151 or ΔC319 and incubated for 36 h. Next, cell lysates were prepared and immunoprecipitated with anti-NSP4 antibodies. Immunoprecipitates were then subjected to western blot analysis using anti-FLAG antibodies. Inputs were probed with anti-FLAG and anti-NSP4 antibodies to confirm protein expression.

On the grounds of previous observations (Figure 4c,e), we hypothesized that the possible interaction between NSP4 and viperin could play a key role in the expulsion of viperin from the ER. To check our hypothesis, HT-29 cells, either mock infected or infected with RV-SA11, were subjected to a co-immunoprecipitation study at 9 hpi. As shown in Figure 5a, NSP4 was observed in the immunoprecipitate of viperin. The absence of VP7, calnexin and COX 4 confirmed that the immunoprecipitate was membrane-free (Figure 5a). A reciprocal co-immunoprecipitation study also confirmed the presence of viperin in the immunoprecipitate of NSP4 (Figure 5b). This was further confirmed, as confocal microscopy revealed the co-localization of viperin and NSP4 (Pearson’s correlation coefficient 0.93) in RV-SA11-infected HT-29 cells at 9 hpi (Figure 5c). Furthermore, NSP4 was transiently co-overexpressed with FLAG-tagged full-length viperin, ∆N-42, ∆N-210, ∆C-151 or ∆C-319 construct in HEK-293 cells. Immunoprecipitation of cell lysates with anti-FLAG antibody confirmed the co-immunoprecipitation of NSP4 with full-length viperin and ∆N-42 but not with ∆N-210, ∆C-151 and ∆C-319 mutants (Figure 5d). Similar results were obtained in the reciprocal co-immunoprecipitation study (Figure 5e). These results suggested that both the radical SAM domain and the C-terminal domain of viperin were necessary for the interaction with NSP4, but the N-terminal domain was not required.

### 3.6. Viperin Inhibited the NSP4-Induced Intrinsic Apoptotic Pathway by Restricting the Translocation of NSP4 to the Mitochondria

**Figure 6:**
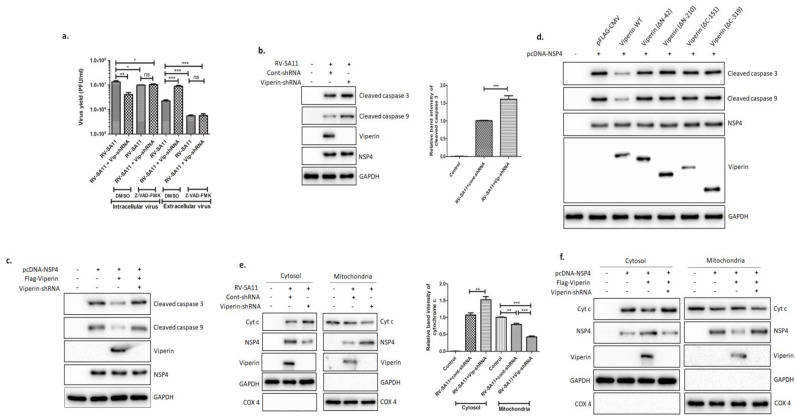
Viperin restricted the RV-induced intrinsic apoptosis pathway by impeding the translocation of NSP4 to the mitochondria. (**a**) HT-29 cells, transfected with either cont-shRNA or vip-shRNA, were infected with RV-SA11 in the presence of DMSO or Z-VAD-FMK (10 µM) and incubated for 24 h. After incubation, both intracellular and extracellular viruses were collected and used to measure viral titers by plaque assay. Data represent mean virus particles ± SD of three independent experiments; *** represents *p* ≤ 0.001, ** represents *p* ≤ 0.01, * represents *p* ≤ 0.05, n represents *p* value non-significant, unpaired Student’s *t*-test. (**b**) Whole cell lysates of RV-SA11-infected HT-29 cells (12 hpi), pre-transfected with either cont-shRNA or vip-shRNA, were assessed by western blot to check expressions of cleaved caspase 3, cleaved caspase 9, viperin, NSP4 and GAPDH. NSP4 and GAPDH served the purpose of the infection marker and the internal loading control, respectively. Relative band intensities of cleaved caspase 3 are also presented. Data represent mean band intensity ± SD of three independent experiments; *** represents *p* ≤ 0.001, unpaired Student’s *t*-test. (**c**) HEK-293 cells were transfected with vectors encoding only NSP4; NSP4 and FLAG-viperin; or NSP4, FLAG-viperin and vip-shRNA. After 36 h of incubation, cell lysates were prepared and subjected to western blot analysis by using anti-cleaved caspase 3, anti-cleaved caspase 9, anti-viperin, anti-NSP4 and anti-GAPDH antibodies. (**d**) HEK-293 cells were transfected with pcDNA-NSP4 along with FLAG-tagged wild-type viperin, ΔN-42, ΔN-210, ΔC-151 or ΔC-319 and incubated for 36 h. Next, cell lysates were prepared and subjected to western blot to check expressions of cleaved caspase 3, cleaved caspase 9, NSP4, viperin and GAPDH. (**e**) HT-29 cells, transfected with either cont-shRNA or vip-shRNA, were infected with RV-SA11 at an MOI of 3 and incubated for 12 h. After incubation, cells were collected and used to isolate cytosolic and mitochondrial fractions. Next, both fractions were subjected to western blot analysis to check expressions of Cyt c, NSP4, viperin, GAPDH and COX 4. COX 4 and GAPDH were used as both the markers and the internal loading controls of the mitochondrial fraction and the cytosolic fraction, respectively. Relative band intensities of cleaved cytochrome c in the cytosolic and mitochondrial fraction are also presented. Data represent mean band intensity ± SD of three independent experiments; *** represents *p* ≤ 0.001, ** represents *p* ≤ 0.01, unpaired Student’s *t*-test. (**f**) Cytosolic and mitochondrial fractions of HEK-293 cells, transfected with vectors encoding only NSP4; NSP4 and FLAG-viperin; or NSP4, FLAG-viperin and vip-shRNA were used to assess the expressions of Cyt c, NSP4, viperin, GAPDH and COX 4 by western blot. COX 4 and GAPDH were used as both the markers and the internal loading controls of the mitochondrial fraction and the cytosolic fraction, respectively.

Given the role of viperin in restricting rotavirus release, we hypothesized that the localization of viperin to the mitochondria might affect the activation of intrinsic apoptosis, previously reported to have a role in rotavirus release [62]. To address this, HT-29 cells, transfected with either cont-shRNA or vip-shRNA, were infected with RV-SA11 in the presence or absence of pan caspase inhibitor Z-VAD-FMK (10 µM), and both intracellular and extracellular rotavirus particles were measured at 24 hpi. Interestingly, in the presence of the caspase inhibitor, no significant difference in intracellular and extracellular viral particles was observed between viperin-knocked-down and viperin-expressing HT-29 cells. However, in DMSO-treated HT-29 cells, a 3.79-fold increase in released virus particles and fold decrease in intracellular virus particles were observed upon viperin knock down (Figure 6a), suggesting that viperin modulated rotavirus release by hindering apoptosis. This was further confirmed when significantly higher cleavage of caspase-9 and caspase-3, hallmarks of intrinsic apoptosis activation, was observed in viperin-knocked-down HT-29 cells compared to viperin-stable HT-29 cells at 12 hpi (Figure 6b). Furthermore, we assessed the consequences of viperin overexpression on the NSP4-induced, intrinsic apoptosis pathway. To perform this, HEK-293 cells were exogenously overexpressed with either NSP4 alone; NSP4 and viperin; or NSP4, viperin and vip-shRNA. After 36 h, the expression of cleaved caspase-3 and cleaved caspase-9 was assessed by immunoblotting. In cells overexpressing viperin, a substantial reduction in the NSP4-induced cleavage of caspase-9 and caspase-3 was observed, whereas increased caspase cleavage was witnessed upon viperin knock down (Figure 6c). Together, these results suggested that viperin restricted NSP4-induced apoptosis during rotavirus infection. Next, to identify the domain of viperin responsible for modulating apoptosis during rotavirus infection, NSP4 and constructs of viperin (WT, ∆N-42, ∆N-210, ∆C-151 and ∆C-319) were co-expressed in HEK-293 cells. Immunoblotting of cell lysates with caspase-9- and caspase-3-specific antibodies revealed that only full-length viperin (WT) effectively inhibited NSP4-induced apoptosis. Cells transfected with either viperin mutants (WT, ∆N-42, ∆N-210, ∆C-151 and ∆C-319) and NSP4 or only NSP4 revealed similar levels of caspase cleavage (Figure 6d). Overall, these results were consistent with the requirement of full-length viperin for anti-rotaviral activity (Figure 3c).

It has previously been reported that NSP4 can integrate into the ER membrane, leading to a rise in cytosolic Ca^2+^ that sequentially activates Bax-mediated intrinsic apoptosis [63]. To address whether viperin-restricted activation of intrinsic apoptosis was due to reduced release of Ca^2+^ into the cytosol, HT-29 cells, transfected with either cont-shRNA or vip-shRNA, were infected with RV-SA11 in the presence of cell-permeant Ca^2+^ chelator BAPTA-AM. Immunoblot analysis of cell lysates revealed increased cleavage of caspase-3 and caspase-9 in viperin-knocked-down cells compared to viperin-expressing cells in the presence of BAPTA-AM (10 µM) at 12 hpi (Figure S4), suggesting that viperin had no effect on the release of Ca^2+^ from the ER to the cytosol. Furthermore, we examined the effect of viperin on the mitochondrial translocation of NSP4 and the cytosolic release of Cyt c. The cytoplasmic and mitochondrial fractions of RV-SA11-infected HT-29 cells, transfected with either cont-shRNA or vip-shRNA, were used to assess the expression of NSP4 and Cyt c by western blot. Compared to viperin-expressing cells, viperin knockdown resulted in increased translocation of NSP4 to the mitochondria and the enhanced release of Cyt c into the cytosol at 12 hpi (Figure 6e). Furthermore, co-overexpression of viperin and NSP4 in HEK-293 cells also ensured reduced translocation of NSP4 to the mitochondria and decreased release of Cyt c into the cytosol compared to only NSP4-expressing cells (Figure 6f). Nevertheless, viperin knockdown restored the mitochondrial level of NSP4 and the cytosolic level of Cyt c in viperin- and NSP4-co-overexpressing HEK-293 cells to an extent comparable to only NSP4-overexpressing HEK-293 cells (Figure 6f). Collectively, these data suggest that viperin modulates the mitochondrial translocation of NSP4 and the subsequent release of Cyt c into cytosol during rotavirus infection.

## 4. Discussion

As an instantaneous response to viral infection, infected cells often induce a dynamic and fundamentally universal innate immune response that leads to the secretion of interferons (IFNs) into the surrounding environment. Recognition of pathogen-associated molecular patterns (PAMPs) by the host pattern recognition receptors (PRRs), notably the retinoic acid-inducible gene I (RIG-I)-like receptors (RLRs), toll-like receptors (TLRs) and nucleotide-oligomerization domain (NOD)-like receptors (NLRs), elicits intricate signaling cascades that result in the expression of IFNs and other early antiviral proteins. Among the three types of IFN, type I IFNs (IFN-α and IFN-β) principally play key roles to establish an antiviral milieu through the activation of the JAK-STAT signaling pathway, which drives the expression of more than 300 interferon-stimulated genes (ISGs). Despite the well-established knowledge regarding the antiviral action of the IFN response, the function of most ISGs remains undetermined. Through in vitro cell culture studies, only a handful of ISGs such as protein kinase R (PKR) [64], 2′-5′-oligoadenylate synthetase (OAS) [65], ISG15 [66], Mx1 [67], viperin [68], etc., have been shown to play antiviral functions against few viruses. Although emerging studies have shed light on the antiviral mechanisms of PKR and OAS during rotavirus infection [69,70], the functional consequence of viperin remains to be elucidated. In this study, we investigated the intricate role of viperin in modulating rotavirus infection. Consistent with previous reports from our lab, an upregulation of viperin protein was observed. The induction of viperin by rotavirus is a strain-independent phenomenon (Figure 1a–e). In virus-invaded cells, viperin can be upregulated by both IFN-dependent and -independent pathways. In the IFN-independent pathway, as described for HCMV, VSV, JEV, HCV and DENV, PAMP-PRR-mediated signaling plays a key role in inducing viperin expression. In contrast, in agreement with the studies on SV, SINV and pseudorabies viruses, we observed that rotavirus induced viperin expression by the type-I IFN dependent pathway, as viperin expression was not observed in rotavirus-infected Vero cells that were deficient in type-I IFN secretion (Figure 1f).

To date, the fundamental molecular mechanism that underprops viperin-arbitrated antiviral action varies among different viruses. Viperin inhibits productive HCMV replication by prohibiting the expression of early–late (pp65), late (gB) and true late (pp28) proteins that are requisite for virus assembly and maturation [30]. Viperin-arbitrated antiviral action against influenza A and HIV-1 is through the inhibition of their egress from the plasma membrane by affecting membrane fluidity and disrupting the lipid rafts [32,44]. For respiratory syncytial virus (RSV), viperin inhibits virus filament formation, leading to impaired virus transmission [71]. In the case of HCV, viperin exerts its antiviral action through interaction with HCV NS5A at the surface of lipid droplets and within the HCV replication complex [38]. The antiviral action of viperin against DENV-2 has been demonstrated to be mediated by its interaction with viral NSP3 and by impeding early viral RNA synthesis [45]. Viperin-restricted replication of ZIKV and tick-borne encephalitis virus (TBEV) is accomplished by targeting NSP3 for proteasomal degradation [71]. Interestingly, JEV counteracts the antiviral action of viperin by targeting it to proteasomal degradation [34]. This diverse array of the antiviral mechanisms of viperin suggests that the functional mode of viperin is multidimensional, and the precise antiviral mechanism is virus-specific. The transition of DLP subviral particles to TLP, a critical step of rotaviral morphogenesis, takes place in the ER. During this process, the DLP subviral particles assembled in the viroplasm bind with NSP4, an integral membrane protein in the ER, through VP6 and bud in to the ER. After the budding process, DLPs acquire a transitory ER membrane envelope containing VP7 and NSP4. These membrane-enveloped particles (MEP) subsequently mature to TLPs by the selective retention of the external capsid proteins VP7 and VP4 and the elimination of NSP4 and membrane lipids [72]. Therefore, primarily we hypothesized that viperin was most likely to have effects on rotaviral morphogenesis. Surprisingly, we found viperin had no profound effect on total infectious virus (TLP) yields during rotavirus infection. Analysis of intracellular and extracellular infectious virus showed viperin knockdown led to augmented viral titer in the extracellular medium and a comparable decrease in the intracellular viral titer of infected host cells, which suggested the involvement of viperin in the release of rotavirus from the infected host cells. We also found an increase in viral RNA in the extracellular medium of infected cells upon viperin knockdown, which was equivalent to increased viral titer in the extracellular medium of viperin-silencing cells. Finally, the kinetic study of extracellular viral release revealed viperin delayed rotavirus egress from the infected host cells (Figure 2).

The specific domain of viperin indispensable for antiviral action differs among viruses and remains undefined for many viruses. Anti-CHKIV activity of viperin is dependent on its ER localization through the N-terminal amphipathic α-helical domain [39]. The central SAM domain is a prerequisite for antiviral action against HIV, TEBV and Bunyamwera virus [44,48,73]. Conversely, the C-terminal domain is essential for the restriction of ZIKV and DENV-2 [45,47]. In addition, both the N-terminal amphipathic helix and the C-terminal residues of viperin are necessary for the restriction of HCV infection, the latter being essential for viperin’s interaction with HCV NS5A [37]. Using a series of deletion mutant constructs of viperin, it was observed that none of the three domains of viperin could independently accomplish anti-rotaviral function; rather, full-length viperin was crucial for efficient antiviral activity (Figure 3).

Viperin is generally localized to the cytosolic face of the ER through its N-terminal amphipathic helix embedded in the ER membrane, where it is believed to play an antiviral role against SINV [31,34], influenza virus [32], HIV [44], HCV [36,74] and HCMV [30]. Interestingly, rotavirus infection triggers intracellular re-localization of viperin from the ER to the mitochondria (Figure 1g–h and Figure 4). Rotaviral NSP4 is recognized as the viral factor that expels viperin from the ER, which can be correlated with the presence of NSP4 in the ER membrane of rotavirus-infected cells [15]. The expulsion of viperin from its normal residence may be a viral strategy to minimize the effects of viperin on rotaviral morphogenesis. During HCMV infection, the subcellular localization of viperin from the ER to the mitochondria by viral protein vMIA has previously been reported [61]. We also evidenced the strong association between NSP4 and viperin by both confocal microscopy and a co-immunoprecipitation study (Figure 5a–c). Co-IP studies involving cells overexpressing NSP4 along with the deletion mutants of viperin revealed the interaction of viperin with NSP4 through its SAM domain and C-terminal domain (Figure 5d,e). Nonetheless, whether this interaction is direct or involves the assistance of other cellular and/or viral factors needs to be further elucidated. The interaction of NSP4 with viperin might play a key role in the expulsion of viperin from the ER membrane.

The role of intrinsic apoptosis in the release of rotavirus from the infected host cells is well-established [22,75]. Given the role of viperin in restricting rotavirus release and its localization to the mitochondria, we hypothesized that virus-induced activation of intrinsic apoptosis might be affected by viperin. Both the titer assay and biochemical study showed that viperin restricted rotavirus release by inhibiting intrinsic apoptosis (Figure 6a,b). NSP4 is the key player that activates intrinsic apoptosis in rotavirus-infected cells [15,22,76]. NSP4-induced cleavage of caspase-9 and caspase-3 was also found to be compromised in HEK-293 cells overexpressing viperin, suggesting the role of viperin in impeding the NSP4-arbitrated intrinsic apoptosis pathway (Figure 6c). Additionally, the deletion mutant study showed that full-length viperin was essential for its anti-apoptotic role (Figure 6d), suggesting that the interaction of viperin with NSP4 via the C-terminal domain and the SAM domain was not sufficient to perform the anti-apoptotic function; rather, the N-terminal domain was also required. These results were consistent with the antiviral function of the N-terminal and C-terminal domains of viperin in preventing rotavirus egress. The integration of viperin into the mitochondrial membrane through its N-terminal domain might be essential for its anti-apoptotic function.

In rotavirus-infected cells, NSP4 can trigger intrinsic apoptosis by two different pathways. First, NSP4, by using its viroporin domain, creates a transmembrane aqueous pore in the ER membrane, leading to the release of Ca^2+^ into the cytosol, which subsequently stimulates Bax-dependent activation of the intrinsic apoptosis pathway [15,75,76]. In the current study, we observed that viperin-restricted activation of intrinsic apoptosis was not due to the reduced release of Ca^2+^ into the cytosol (Figure S4). Second, NSP4 could mediate Bax-independent, pro-apoptotic function by translocating to the mitochondria. NSP4 results in the dissipation of mitochondrial reduction potential and the subsequent release of Cyt c into the cytosol, leading to the activation of intrinsic apoptosis [22,62]. In the present study, viperin knockdown boosted translocation of NSP4 to the mitochondria, resulting in increased release of Cyt c into the cytosol and heightened activation of the intrinsic apoptosis pathway, whereas viperin overexpression resulted in reduced translocation of NSP4 to the mitochondria and the subsequent reduced release of Cyt c into the cytosol in HEK-293 cells (Figure 6e,f). Thus, our study highlighted the intricate modulation of host antiviral IGS by a viral protein and its impact on virus-induced apoptosis.

In conclusion, the study highlights the underlying molecular mechanism by which viperin exerts its antiviral action during rotavirus infection. We propose that rotaviral NSP4 drives the relocalization of viperin from the ER to the mitochondria, where viperin, in turn, limits the release of Cyt c into the cytosol, resulting in the inhibition of intrinsic apoptosis to restrict rotavirus release. Therefore, our study delineates a novel stratagem adopted by the host to restrict rotavirus egress and uncovers a new dynamic of virus–viperin interaction, which sheds light on the intricate relationship between host and viral proteins for successful infection.

## Figures and Tables

**Table 1:** List of primers used for the study.

Gene	Primers	Restriction Enzyme	Expression Vector
Viperin (wild type)	Forward: 5’-GCCGCGAATTCAGATGTGGGTGCTTACACCTGCT-3′	EcoR1	pFLAG-CMV6b (Sigma)
	Reverse: 5’-ATTAGGTCGACGTAGCAGCCAGAAGGTTGCCCT-3′	Sal1	
Viperin (ΔN-42)	Forward: 5’-GCCGCGAATTCAGGCTACCAAGAGGAGAAAGCA-3′	EcoR1	pFLAG-CMV6b (Sigma)
	Reverse: 5’-ATTAGGTCGACGTAGCAGCCAGAAGGTTGCCCT-3′	Sal1	
Viperin (ΔN-210)	Forward: 5’-ATCCGGTCGACGCTACCAATCCAGCTTCAGATCA-3′	EcoR1	pFLAG-CMV6b (Sigma)
	Reverse: 5’-ATTAGGTCGACGTAGCAGCCAGAAGGTTGCCCT-3′	Sal1	
Viperin (ΔC-151)	Forward: 5’-GCCGCGAATTCAGATGTGGGTGCTTACACCTGCT-3′	EcoR1	pFLAG-CMV6b (Sigma)
	Reverse: 5’-ATCAGGTCGACGCCACCTCCTCAGCTTTTGAAGG-3′	Sal1	
Viperin (ΔC-319)	Forward: 5’-GCCGCGAATTCAGATGTGGGTGCTTACACCTGCT-3′	EcoR1	pFLAG-CMV6b (Sigma)
	Reverse: 5′-ATCAGGTCGACGCCACCTCCTCAGCTTTTGAAGG-3′	Sal1	
NSP4	Forward: 5′-GAATTCATGGAAAAGCTTACCGACC-3′	EcoR1	pcDNA^TM^6/V5-His B (Invitrogen)
	Reverse: 5′-GATATCCACATTGCTGCAGTCACTTCT-3′	EcoRV	

## Data Availability

All data contained within this manuscript are available upon reasonable request from the corresponding author.

## Supplementary Materials

The following are available online at https://www.mdpi.com/article/10.3390/v13071324/s1, Figure S1: Flowchart diagram of the study design. Figure S2: Cell viability analysis of viperin-knocked-down cells. Figure S3: Cell viability analysis of HEK-293 cells overexpressing WT-viperin or mutant viperin. Figure S4: Cytoplasmic release of Ca^2+^ from ER is not influenced by viperin during RV infection.
