# Supplementary material for: Viperin, an IFN-Stimulated Protein, Delays Rotavirus Release by Inhibiting Non-Structural Protein 4 (NSP4)-Induced Intrinsic Apoptosis
Source: Viruses. 2021 Jul 8;13(7):1324. doi: 10.3390/v13071324 (PMC8310278; doi:10.3390/v13071324)
Supplement: Supplementary file 1 [file viruses-13-01324-s001.zip › viruses-1174853-supplementary.pdf]

viruses

MDPI

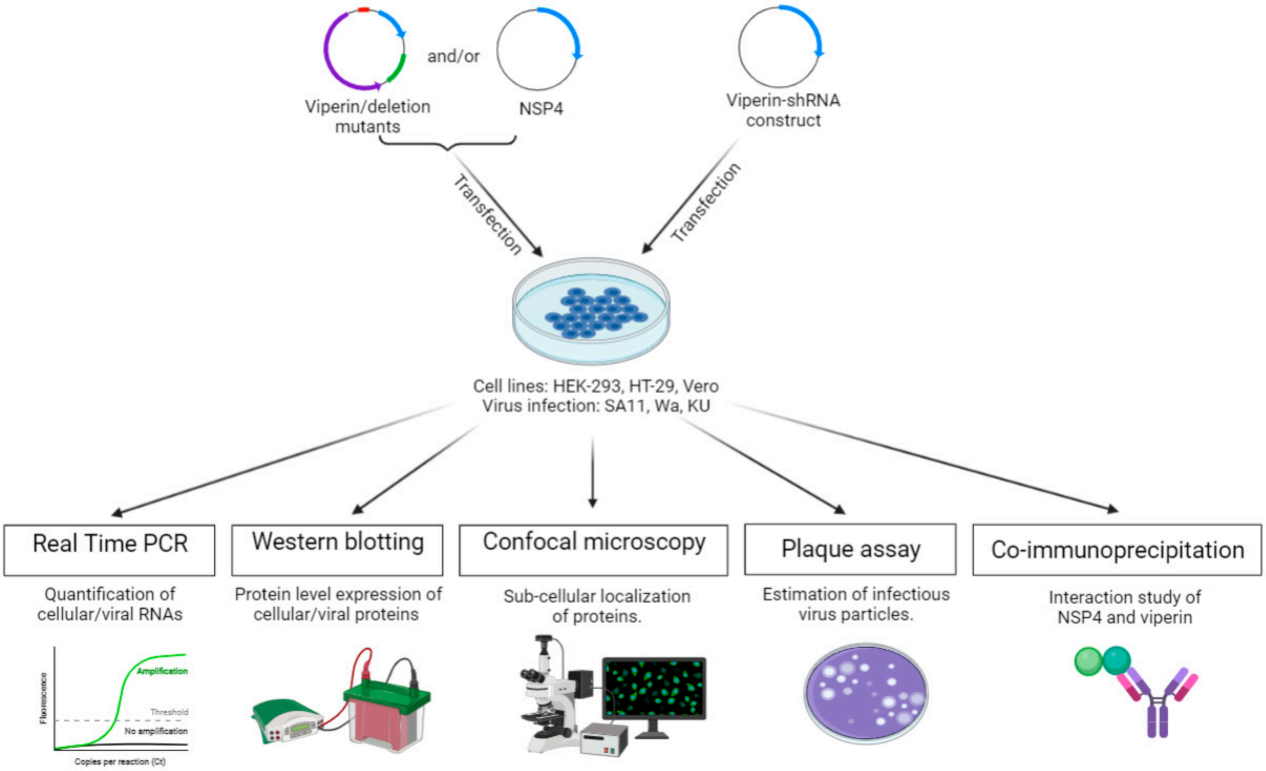

Figure S1. Flowchart diagram of the study design.

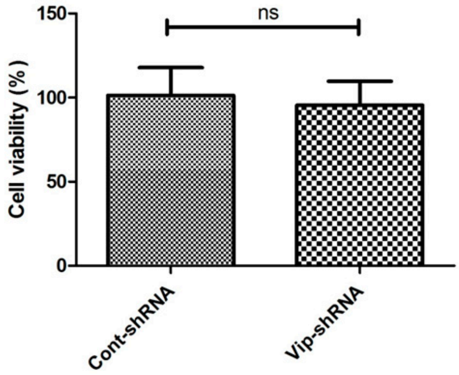

Figure S2. Cell viability analysis of viperin knocked down cells. HT-29 cells were transfected with either cont-shRNA or vip-shRNA and incubated for 72 hours. After incubation, cells were subjected to MTT assay to check cell viability. Data was represented as mean ± SD of three independent experiments. ns represents *P* value not significant, unpaired student's t test.

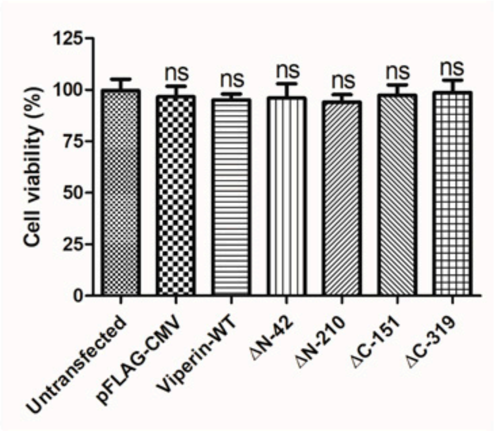

Figure S3. Cell viability analysis of HEK-293 cells overexpressing WT-viperin or mutant viperin**.** HEK-293 cells were transfected with either control vector or vector encoding WT-viperin, ΔN-42 viperin, ΔN-210 viperin, ΔC-151 viperin or ΔC-319 viperin and incubated for 72 hrs. After incubation, cells were subjected to MTT assay to check cell viability. Data was represented as mean ± SD of three independent experiments. ns represents *P* value not significant, unpaired student's t test.

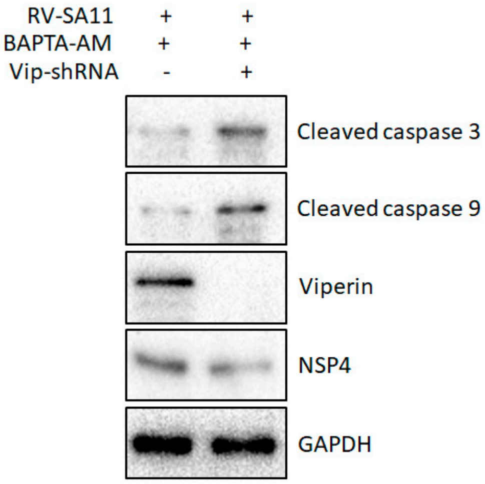

Figure 4. Cytoplasmic release of Ca^{2+} from ER is not influenced by viperin during RV infection. HT-29 cells, transfected with either cont-shRNA or vip-shRNA, were infected with RV-SA11 at an MOI of 3 in the presence of cell permeable chelator BAPTA-AM (50 µM) and incubated for 9 hrs. Next, cell lysates were prepared and subjected to western blot analysis using antibodies specific for cleaved caspase 3, cleaved caspase 9, viperin, NSP4 and GAPDH.
